# The electromigration effect revisited: non-uniform local tensile stress-driven diffusion

**DOI:** 10.1038/s41598-017-03324-5

**Published:** 2017-06-08

**Authors:** Shih-kang Lin, Yu-chen Liu, Shang-Jui Chiu, Yen-Ting Liu, Hsin-Yi Lee

**Affiliations:** 1 Department of Materials Science and Engineering, National Cheng Kung University, Tainan city, 70101 Taiwan; 2 Center for Micro/Nano Science and Technology, National Cheng Kung University, Tainan city, 70101 Taiwan; 3 National Synchrotron Radiation Research Center, Hsinchu city, 30076 Taiwan

## Abstract

The electromigration (EM) effect involves atomic diffusion of metals under current stressing. Recent theories of EM are based on the unbalanced electrostatic and electron-wind forces exerted on metal ions. However, none of these models have coupled the EM effect and lattice stability. Here, we performed *in situ* current-stressing experiments for pure Cu strips using synchrotron X-ray diffractometry and scanning electron microscopy and *ab initio* calculations based on density functional theory. An intrinsic and non-uniform lattice expansion – larger at the cathode and smaller at the anode, is identified induced by the flow of electrons. If this electron flow-induced strain is small, it causes an elastic deformation; while if it is larger than the yield point, diffusion as local stress relaxation will cause the formation of hillocks and voids as well as EM-induced failure. The fundamental driving force for the electromigration effect is elucidated and validated with experiments.

## Introduction

The electromigration (EM) effect describes atomic diffusion in conductors driven by electric currents, which may lead to the formation of voids and hillocks at the cathode and the anode, respectively^1^. The driving force of EM is generally interpreted by the semi-ballistic model based on momentum transfers between electrons and ions in free electron gas^2^. A more advanced model taking the complexity of band structure of materials into account, namely the polarization model, is proposed, where the electric current-induced force is suggested to be the sum of the external electrostatic force and the electron perturbation-induced force^3, 4^. The existing theories successfully address the *directional effects* of EM-induced phenomena, which are strongly correlated with the directions of electron or electric current flows^5^. However, some intriguing phenomena beyond the *directional effects*, namely, the *non-directional effects*, cannot be explained with the “forces” proposed in either of the above mentioned models. These *non-directional* EM-induced phenomena involve supersaturation of alloys^6^, the non-polarity effect of IMC growth in interfacial reactions^7^, and crystallographic changes, such as electrorecrystallization^8^, grain rotation^9^, diminishing of crystal structure^10^, and dislocation generation^11–14^. Lin *et al*.^15^ recently modeled the electric current-induced excess Gibbs free energy using the *ab initio*-aided CALPHAD method, and successfully predicted the phase relations between the face-centered cubic (fcc) and the body-centered tetragonal (bct) phases in the Pb-Sn system under electric current stressing. However, that was a semi-empirical model based on experimentally measured rates of Sn whisker growth driven by external stresses and electric currents^1, 16^. Nevertheless, the semi-empirical model successfully rationalized the *non-directional* EM-induced supersaturation of alloys, which leads to a fundamental question: What are the effects of electric currents upon materials lattice stability, which is independent of the direction of the electron flows?

In this work, a combinatorial approach of *ab initio* calculations based on density functional theory (DFT) and systematical *in situ* current-stressing experiments with synchrotron X-ray diffraction (XRD) and scanning electron microscopy (SEM) was performed to study the effects of electrical currents upon the lattice stability of pure Cu as a model material. The theoretical calculations agree closely with the experiments, opening the door for new understandings of the peculiar *non-directional* phenomena under electric current stressing, the analogy of materials responses under electric current stressing and conventional mechanical stress, and the fundamental driving force of EM.

## Methods

### Preparation dog-bone shaped specimens

**Figure 1:**
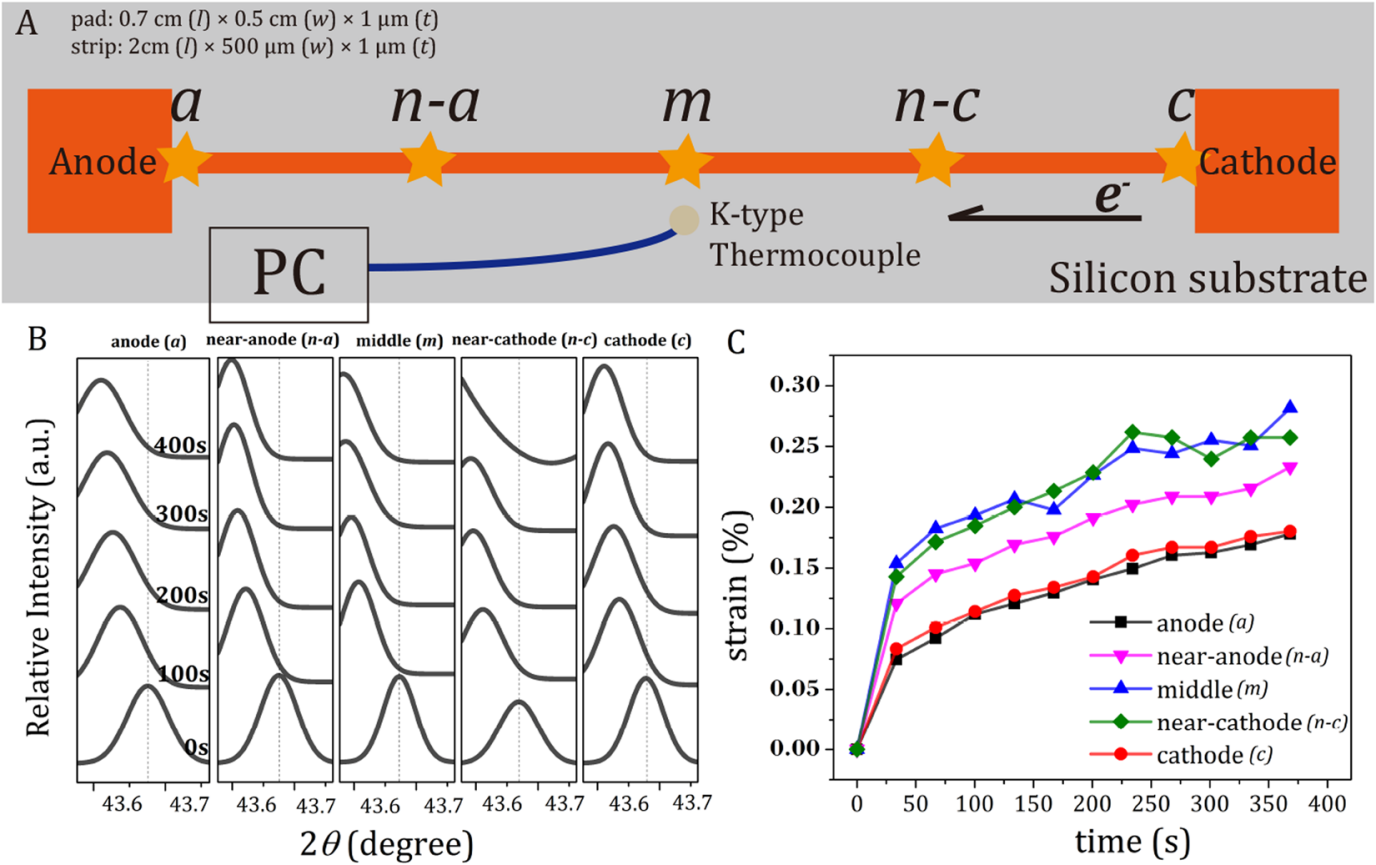
(**A**) The schematic diagram of the dog-bone-shape pure Cu samples. Five positions along the Cu strip, namely the “anode” (*a*), “near anode” (*n-a*), “middle” (*m*), “near cathode” (*n-c*), and “cathode” (*c*), were characterized using synchrotron X-ray diffraction. A *K*-type thermal couple was attached by the center of Cu strip for monitoring the temperature of Cu strip during current stressing. (**B**) The XRD peaks of the Cu-(111) plane detected at the five positions of the Cu strip during current stressing at the current density of 4 × 10^5^ A/cm^2^. (**C**) The translated strain evolutions of the Cu-(111) plane during current stressing at the five positions of the Cu strip.

One μm-thick Cu film was deposited on a TaN-coated high-resistivity Si wafer by a radio frequency (RF) balanced magnetron sputtering system with the power of 300 W and deposition rate of 105 Å/min. under a vacuum of 3 × 10^−3^ torr. The dog-bone structure was patterned on the Cu/TaN/Si wafer using a laser-cut hard mask. As shown in Fig. 1A, the size of each pad is 0.5 cm × 0.7 cm, while the width of the strips is 2 cm in length with various widths of 50 μm, 80 μm, 100 μm, and 500 μm. The dog-bone specimens were annealed at 400 °C for 2 h under N_2_ atmosphere to eliminate the intrinsic stresses prior to current-stressing experiments. (see Fig. S1 in supplementary text for the Cu *kα* X-ray diffraction (XRD) pattern of the annealed samples indexed with the JCPDS database (PDF #65-9743)). These fully relaxed Cu dog-bone specimens were subjected to *in situ* current-stressing experiments.

### *In situ* synchrotron X-ray diffraction and scanning electron microscopy

*In situ* current-stressing experiments with synchrotron XRD measurements were performed using the beam-line 17B1 of the National Synchrotron Radiation Research Center (NSRRC) in Hsinchu, Taiwan. Goniometer of Huber 8-circle diffractometer was used (see Fig. S2A in supplementary text for the experimental setup of the *in situ* current-stressing experiments). The sample was placed on a Al_2_O_3_ holder and clapped with Cu electrodes at both pads of the dog-bone specimen. The dimension for Al_2_O_3_ stage was 9 cm × 1 cm × 1 cm. The dimension for the Cu electrodes was 2 cm × 1 cm × 3 mm. The X-ray energy was 8000 eV with the spot size of 900 × 900 μm^2^. The scan rate and step size of measurements were 0.024°/s and 0.012°, respectively. A fresh and fully relaxed Cu strip was used for each measurement. In addition, *in situ* XRD experiments were also performed for samples under thermal aging without current stressing, as control experiments to differentiate the effects induced by electric currents and heat on crystal structures. Microstructures of the samples under electric current stressing were also *in situ* examined using a customized scanning electron microscope (SEM, Hitachi SU3500, Japan) with a 10 keV acceleration voltage (see Fig. S2B in supplementary text for the experimental setup of the *in situ* current-stressing experiments, where the same Al_2_O_3_ holder and Cu electrodes were used). External power supply was used through the port to perform the electric current experiment.

### Finite-element analysis

The finite-element analysis was performed using the ANSYS software. The model for a dog-bone shaped Cu specimen on a silicon substrate was constructed. The dimension for the Cu dog-bone shaped specimen was exactly as that in experiment, while the dimension for the silicon substrate was 3.6 cm × 0.7 cm × 1 mm. The silicon substrate was placed on a Al_2_O_3_ stage and clapped by two Cu electrodes at both the edge sides, as the setup in experiments. The dimension for Al_2_O_3_ holder and the two Cu electrodes is aforementioned. The simulations were performed with the whole sample embedded in air and the initial temperature was 30 °C. Thermal conduction was allowed at the interfaces between the silicon substrate and the Al_2_O_3_ stage, as well as between the silicon substrate and the two Cu electrodes. The thermal radiations from all the surfaces were also allowed. The coefficient of convection was 5 Wm^−2^ K^−1^. The Joule heating-induced temperature profile was obtained by applying voltage upon the two Cu electrodes to reach the current density of 4 × 10^5^ A/cm^2^.

### *Ab initio* calculations

The Vienna *Ab-initio* Simulation Package (VASP)^17^, based on the density functional theory (DFT) with a plane wave basis, was employed to simulate the electron perturbation of pure Cu. Generalized gradient approximation (GGA) exchange-correlation functional parameterized by Perdew-Burke-Ernzerhof (PBE)^18^ and projector augmented wave (PAW) method^19^ with an energy cut-off of 410 eV were used. 6 × 6 × 6 *k*-point meshes for Brillouin zone sampling using Monkhost-Pack scheme^20^ were performed for the 32-atom Cu supercell. The numerical integration of the Brillouin zone and the energy cut-off were verified to produce absolute energy convergence to better than 10^−4^ eV/atom, with the forces at each atomic site converged to within 10^−3^ eV/Å.

## Results

### Electric current-induced intrinsic non-uniform lattice expansion

Figure 1A shows the schematic diagram of the dog-bone shaped pure Cu sample with 1 μm-thick (*t*), 500 μm-wide (*w*), and 2 cm-long (*l*) strip and a large pad as the electrode on each side for *in situ* current-stressing experiments at the current density of 4 × 10^5^ A/cm^2^. Five positions along the Cu strip, namely the “anode” by the anode pad, “near anode” at the quarter of strip from the anode pad, “middle” at the center of strip, “near cathode” at the quarter of strip from the cathode pad, and “cathode” by the cathode pad, respectively, were *in situ* characterized using synchrotron X-ray diffraction. As shown in Fig. 1B, the characteristic peaks of the (111) plane at all the five positions on the Cu strip gradually shifted toward the low-angle side, indicating the Cu lattice expanded during current stressing. To quantify the extent of lattice expansion, the peak shifting was translated into the strain (*ε*) along the (111)-plane normal according to Eq. (1):

1 $$\varepsilon =\frac{d-{d}_{0}}{{d}_{0}}\times 100 \% ,$$

where *d* and *d*_0_ are the measured and equilibrium *d*-spacing of the (111) plane of Cu derived based on Bragg’s law, respectively. Figure 1C shows the strain evolutions of the (111) plane of Cu during current stressing at the “anode”, “near-anode”, “middle”, “near-cathode”, and “cathode” of the strip, respectively. Surprisingly, tensile strains were found at all positions of the Cu strip, contradicting the conventional EM theories in the literature that materials would be expanded at the cathode and compressed at the anode under current stressing^11–14, 21, 22^. The tensile strains at the five points were generated instantly at the early stage of current stressing and then gradually increased at a similar rate. Moreover, the extents of the initial lattice expansion under current stressing were not equal at the five positions – the largest expansion at the “middle” and “near cathode”, a medium expansion at the “near anode”, and the smallest expansion at the “cathode” and “anode”. The spatial distribution of tensile strain is not symmetrical to the dog-bone specimen, nor sequenced in the order followed by electron flow. A non-uniform tensile strain and its evolution are revealed for pure Cu under electric current stressing.

**Figure 2:**
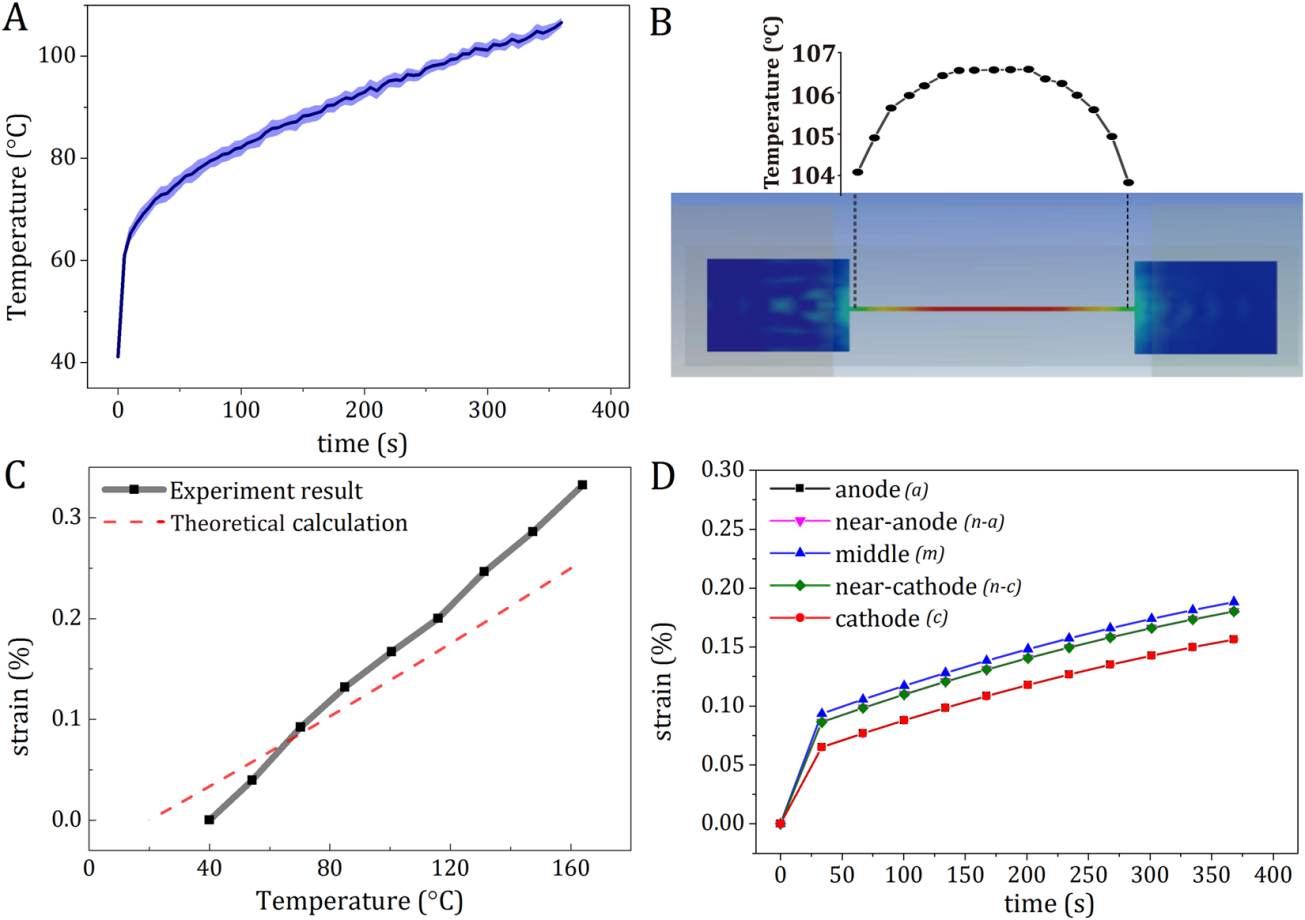
(**A**) The temperature evolution of Cu strips under current stressing at the current density of 4 × 10^5^ A/cm^2^. (**B**) The finite-element analysis of temperature distribution along the Cu strip induced by Joule heating at the current density of 4 × 10^5^ A/cm^2^. (**C**) The *in situ* thermal expansion-induced strain (black solid line) of the Cu (111) plane and the derived strain (red dashed line) based on the coefficient of thermal expansion of bulk Cu against temperature. (**D**) The derived Joule heating-induced strain evolutions of the “anode”, “near anode”, “middle”, “near cathode”, and “cathode” of the Cu strip under current stressing at the current density of 4 × 10^5^ A/cm^2^.

Since the joule heating during current stressing would induce thermal expansion, it must be carefully calibrated to evaluate the sources of the non-uniform tensile strain under current stressing. The temperature of the Cu strip under current stressing was determined by an *in situ* measurement using a thermal couple attached to the center of the Cu strip (Fig. 1A), and a finite-element simulation, as shown in Fig. 2A and B, respectively. As expected, the temperature of the Cu strip gradually increased under current stressing, and it reached a value of 105.5 °C at 350 s as shown in Fig. 2A. The finite-element simulation reveals the microscopic temperature distribution along the Cu strip under current stressing. As shown in Fig. 2B, a symmetrical temperature distribution in sinusoidal form along the Cu strip was exhibited with a maximum of 106.3 °C at the middle and a minimum of 103.5 °C at the ends near the two pads. The simulation agrees closely with the measurements, suggesting the thermal expansion developed with time, while it distributed symmetrically along the strip with a negligible temperature difference of less than 3 °C. To quantitatively evaluate the thermal expansion, *in situ* thermal aging with synchrotron X-ray diffraction was performed for temperatures ranging from 40 to 160 °C, as shown in Fig. 2C. The measured thermal expansion-induced strain (black solid line) of the Cu (111) plane exhibits a linear trend in the same order of magnitude of the derived strain (red dashed line) against temperature based on the coefficient of thermal expansion of bulk Cu^23^. This discrepancy of the measured and derived thermal expansion-induced strains is likely due to the geometry of the thin-film Cu strip as well as the substrate effect. Based on the *in situ* temperature measurements under current stressing (Fig. 2A), finite-element simulation (Fig. 2B), and *in situ* thermal expansion-induced strain measurements (Fig. 2C), the strain evolution of the Cu strip solely caused by Joule heating under current stressing at the “anode”, “near anode”, “middle”, “near cathode”, and “cathode” can be deduced, as shown in Fig. 2D.

Hence, the true electron flow-induced strain (*ε*_electron_) could be extracted by subtracting the total strain (*ε*_total_) shown in Fig. 1C by the thermal expansion-induced strain (*ε*_Joule heating_) shown in Fig. 2D, as given in Eq. (2):

2 $${\varepsilon }_{{\rm{t}}{\rm{o}}{\rm{t}}{\rm{a}}{\rm{l}}}={\varepsilon }_{{\rm{J}}{\rm{o}}{\rm{u}}{\rm{l}}{\rm{e}}{\rm{h}}{\rm{e}}{\rm{a}}{\rm{t}}{\rm{i}}{\rm{n}}{\rm{g}}}+{\varepsilon }_{{\rm{e}}{\rm{l}}{\rm{e}}{\rm{c}}{\rm{t}}{\rm{r}}{\rm{o}}{\rm{n}}}$$

**Figure 3:**
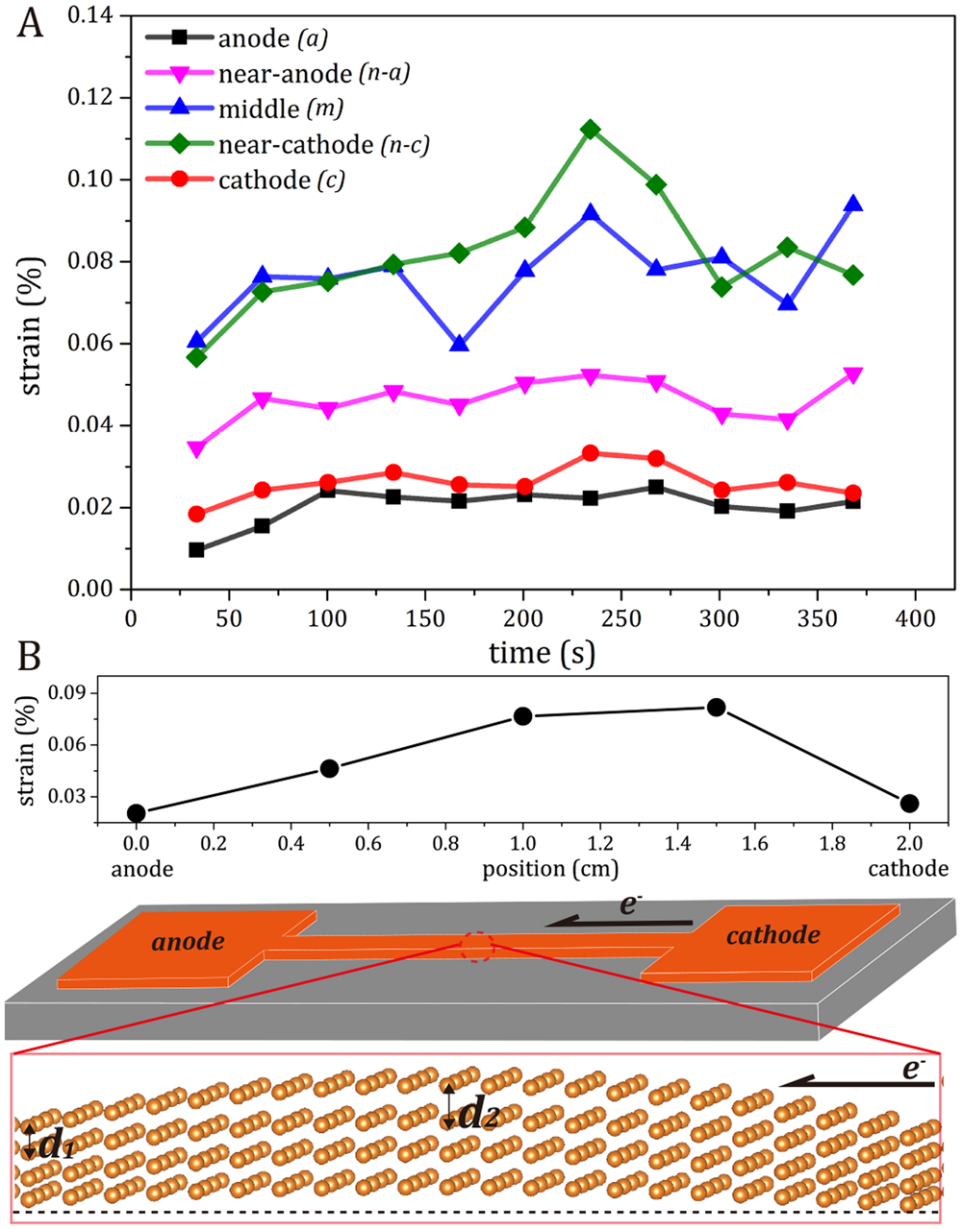
(**A**) The true electron flow-induced strains evolution of the “anode” (*a*), “near anode” (*n-a*), “middle” (*m*), “near cathode” (*n-c*), and “cathode” (*c*) of Cu strip under stressing at the current density of 4 × 10^5^ A/cm^2^ (**B**) The extents of electron flow-induced strains along the Cu strip, and the schematic diagram illustrating the constrained electron flow-induced lattice expansion of the dog-bone-shape Cu sample.

As shown in Fig. 3A, the true electron flow-induced strains at all the five positions of the Cu strip essentially remained unchanged during current stressing. The result is not that surprising because the experiments were performed at a given current density; however, it was very interesting to find the extents of electron flow-induced strains varied along the Cu strip – smallest strains at the “anode” and the “cathode”, medium strains at the “near-anode”, and largest strains at the “near-cathode” and the “middle”. Presumably, all the true electron flow-induced strains (*ε*_electron_) resulted from the flow of electrons at a high current density (4 × 10^5^ A/cm^2^), so a much smaller electron flow-induced expansion is expected at the two pads, where the current density is an order of magnitude lower (4 × 10^4^ A/cm^2^). As illustrated in Fig. 3B, it is intuitively comprehensible that the stiffness of Cu varies along the strip due to the geometry of the dog-bone-shape specimen, *i.e*., the two pads constrain the expansion of the neighboring strip at the “anode” and the “cathode”, while the maximum strain at the “middle” is the most unconstrained. However, the “near-cathode” and the “near-anode” are at symmetrical positions with, in principle, identical stiffness upon electron flow-induced expansion, but a larger strain was detected at the “near-cathode” and a smaller strain was found at the “near-anode”, leading to a tensile strain gradient along the flow of electrons. The non-uniform deformation induced by electric currents was also reported in Al^12^ and Cu^11^ system based on synchrotron-based Laue diffraction. Therefore, if there were no constraint from the dog-bone-shape specimen, presumably the maximal and minimal strains should occur at the cathode and the anode, respectively, with a descending trend along the Cu strip. In summary, the intrinsic electric current-induced strain is identified, establishing a tensile-strain gradient from the cathode-side toward the anode-side during current stressing.

### Electron perturbation-induced lattice expansion

**Figure 4:**
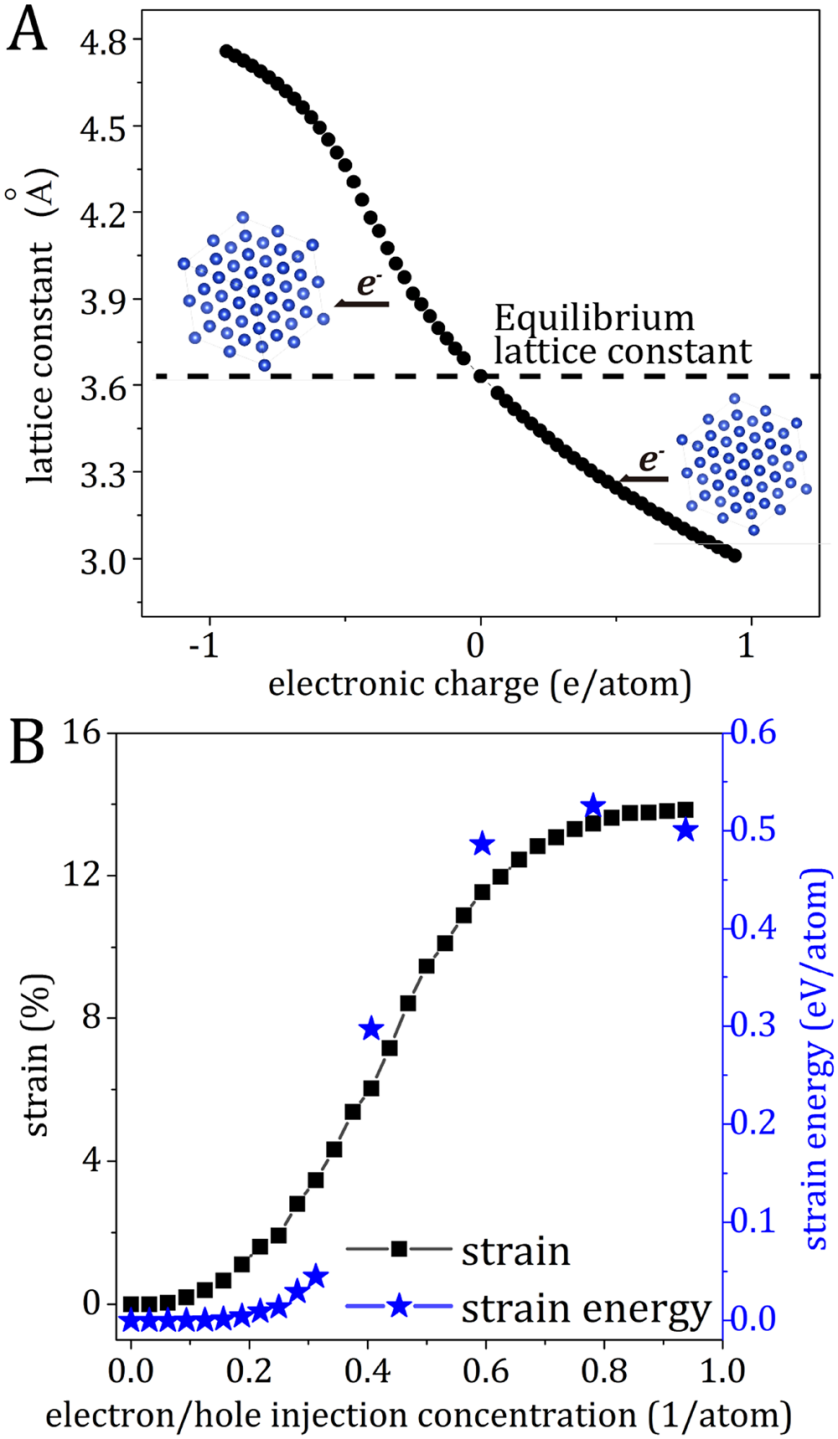
(**A**) *Ab initio* lattice constant of Cu with electron injection (negative charges) or ejection (positive charges) and (**B**) The net *ab initio* tensile strain and corresponding strain energy of Cu against of the extent of electron perturbation.

To investigate the origin of the intrinsic electric current-induced strain and the strain gradient during current stressing, *ab initio* calculations were performed to simulate the flow of electrons using two separate charge-compensated supercells with either extra electron(s) or hole(s). As shown in Fig. 4A, with the electron (negative charges) and hole (positive charges) injection, the Cu lattice would be expanded and contracted, respectively. Interestingly, at the same electron- and hole-injection level, the extents of expansion and contraction of the Cu lattice differ – a larger expansion with an electron injection and a smaller contraction with a hole injection. Hence, when electrons enter from a neighboring atom (unit cell) and exit to the other neighboring atom (unit cell), the electron perturbation during the electron flow will yield a net tensile strain (*ε*_electron_), as shown in Fig. 4B. The maximum extent of electron/hole injection can be estimated by assuming each free electron from each Cu atom simultaneously participates in the electron perturbation, *i.e*., 1 (1/atom) or ca. 8.5 × 10^28^ (1/m^3^) based on the free electron model^24^. Note that the calculations were done in a freestanding system without constraints from boundary conditions, so overestimated strains were anticipated. Nevertheless, the results clearly show the electron perturbation can induce intrinsic tensile strain, and a large extent of electron perturbation leads to a large net tensile strain and a large corresponding strain energy. This provides a direct implication to explain the occurrence of strain gradient under current stressing from the cathode toward the anode as shown in Fig. 3B, that is, electrons at the cathode-side may induce a larger extent of electron perturbation, leading to larger net tensile strains and larger strain energies, so a strain gradient (Δ*ε*_electron_) or a strain-energy gradient (Δ*U*) is established. The *ab initio* calculations closely agree with the *in situ* experiments.

### Electric current-induced “yield strain” and atomic diffusion

**Figure 5:**
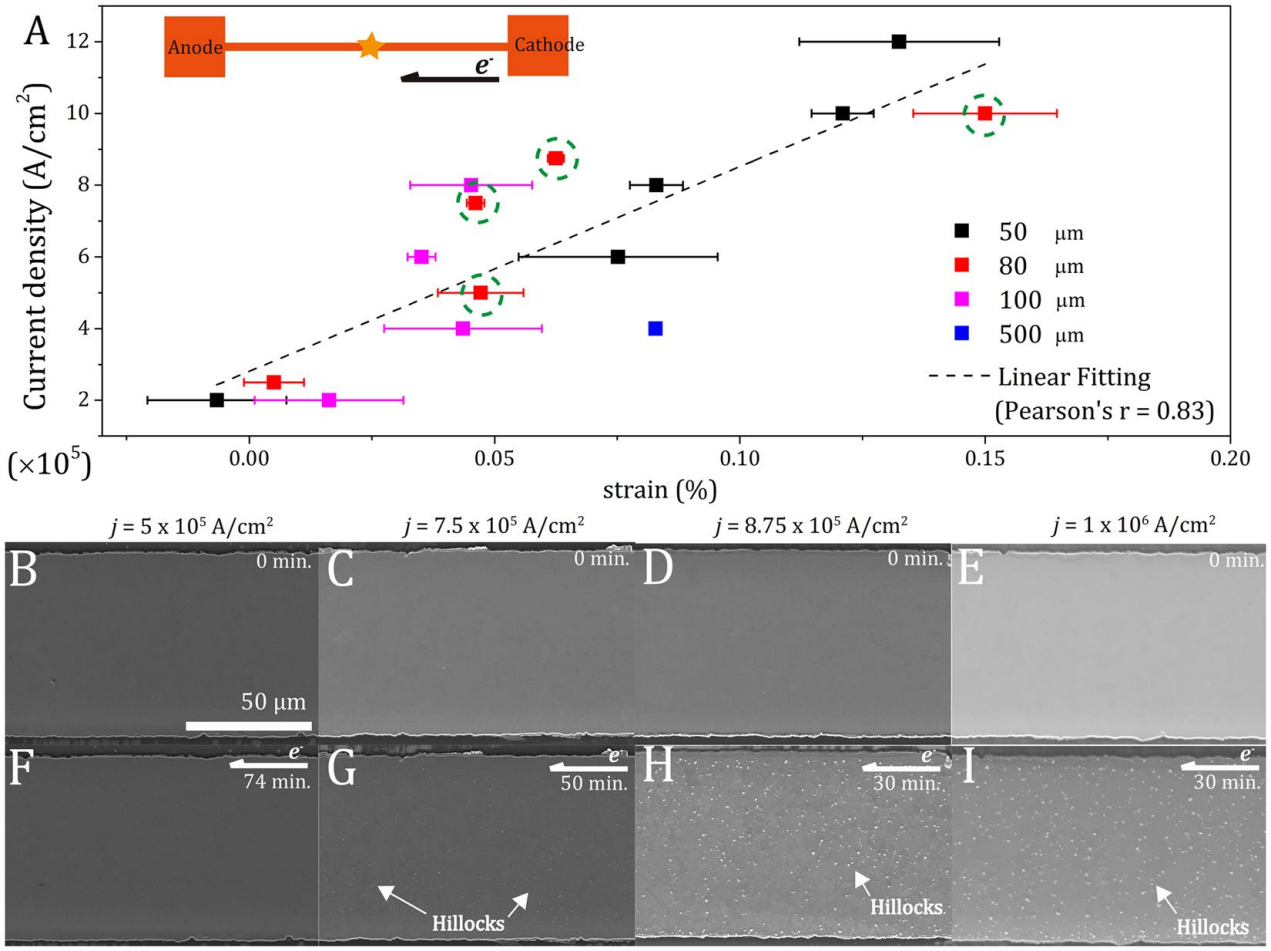
(**A**) The electron flow-induced strains of the 50, 80, 100, and 500 μm-wide Cu strips under current stressing at various current densities. The Pearson correlation coefficient, r, for the linear fitting is 0.83. The SEIs of the 80 μm-wide Cu strips (**B**–**E**) prior to current stressing and under current stressing at the current density of (**F**) 5.0 × 10^5^ A/cm^2^ for 74 min., (**G**) 7.5 × 10^5^ A/cm^2^ for 50 min., (**H**) 8.75 × 10^5^ A/cm^2^ for 30 min., and (**I**) 1.0 × 10^6^ A/cm^2^ for 30 min.

To evaluate the effect of current density upon the aforementioned electron flow-induced strain, narrower specimens were subjected to the *in situ* current-stressing experiments at current densities ranging from 2.0 × 10^5^ to 1.2 × 10^6^ A/cm^2^. Since the resistances and current densities vary among the specimens, different extents of electric current-induced Joule heating and the induced thermal expansion were expected. As in the aforementioned procedure, the true electron flow-induced strains can be derived by subtracting the total *in situ* strains under current stressing by the corresponding thermal strains. Figure 5A shows the electron flow-induced strains at various current densities derived from the *in situ* synchrotron X-ray and temperature measurements at the center of the 50, 80, 100, and 500 μm-wide Cu strips (see supplementary text and Fig. S3–S8 for the total strains and the details of derivations and data processing for obtaining the electron flow-induced strains). Although the electron flow-induced strain is largely affected by the grain structure of an individual specimen and the local distribution of electric current, such as current crowding, a highly proportional linear trend between the current density and electron flow-induced strain is obtained. As expected, a higher current density involves a larger extent of electron perturbation and consequently a larger electron flow-induced tensile strain.

Figure 5B–E and F–I show the *in situ* secondary electron images (SEIs) of the 80 μm-wide Cu strips before and after current stressing at various current densities ranging from 5.0 × 10^5^ to 1.0 × 10^6^ A/cm^2^, as those marked with dashed green circles in Fig. 5A, respectively. As shown in Fig. 5B–E, a clean and smooth surface can be seen at all the specimens prior to current-stressing experiments. After a 74 min.-current stressing at the lowest current density (5.0 × 10^5^ A/cm^2^) as shown in Fig. 5F, no microstructural changes, such as the formation of hillocks and voids, can be found. Its voltage-read also remained steady during the experiment. However, for specimens after current stressing at the higher current densities of 8.75 × 10^5^ and 1.0 × 10^6^ A/cm^2^ for 30 min. as shown in Fig. 5H and I, respectively, an extensive formation of hillocks can be seen, indicating the EM occurred. For the specimen experimented at the medium current density between the two extreme cases, *i.e*., 7.5 × 10^5^ A/cm^2^, hillocks started to form after current stressing for ca. 30 min. (see Fig. S9 in supplementary for the morphology evolution), while the voltage-read gradually increased with the greater resistance associated with the microstructural changes. Its microstructure after current stressing for 50 min. is shown in Fig. 5G. The size and density of the hillocks are significantly smaller than those found in the samples experimented at 8.75 × 10^5^ or 1.0 × 10^6^ A/cm^2^ for 30 min., as shown in Fig. 5H and I, respectively.

In summary, the electron flow-induced strain highly correlates with the microstructural changes after current stressing. A larger tensile strain is induced by the flow of electrons at a higher current density, as shown in Fig. 5A. Based on these results shown in Fig. 5B–I, a critical, or a “yield”, electron flow-induced strain exists – the “yield strain” is anticipated when the strain energy and the displacement between the lattice planes are sufficiently large to trigger a stress relaxation enabled by atomic diffusion. Below this “yield strain”, the electron flow-induced strains as well as the strain gradients do not cause diffusion or plastic deformation during current stressing. Instead, the tensile strain can be relaxed reversibly after the cessation of current stressing as an elastic deformation in the conventional solid mechanics. On the contrary, diffusion and plastic deformation will occur for the specimens experimented on at conditions beyond the “yield strain”. This non-uniform electron flow-induced strain is associated with an unbalanced stress to provide the driving force for diffusion based on the diffusional creep mechanism^25^, *i.e*., the driving force for the EM. The context of electron flow-induced strain and its diffusional relaxation can be analogized to conventional solid mechanics.

**Figure 6:**
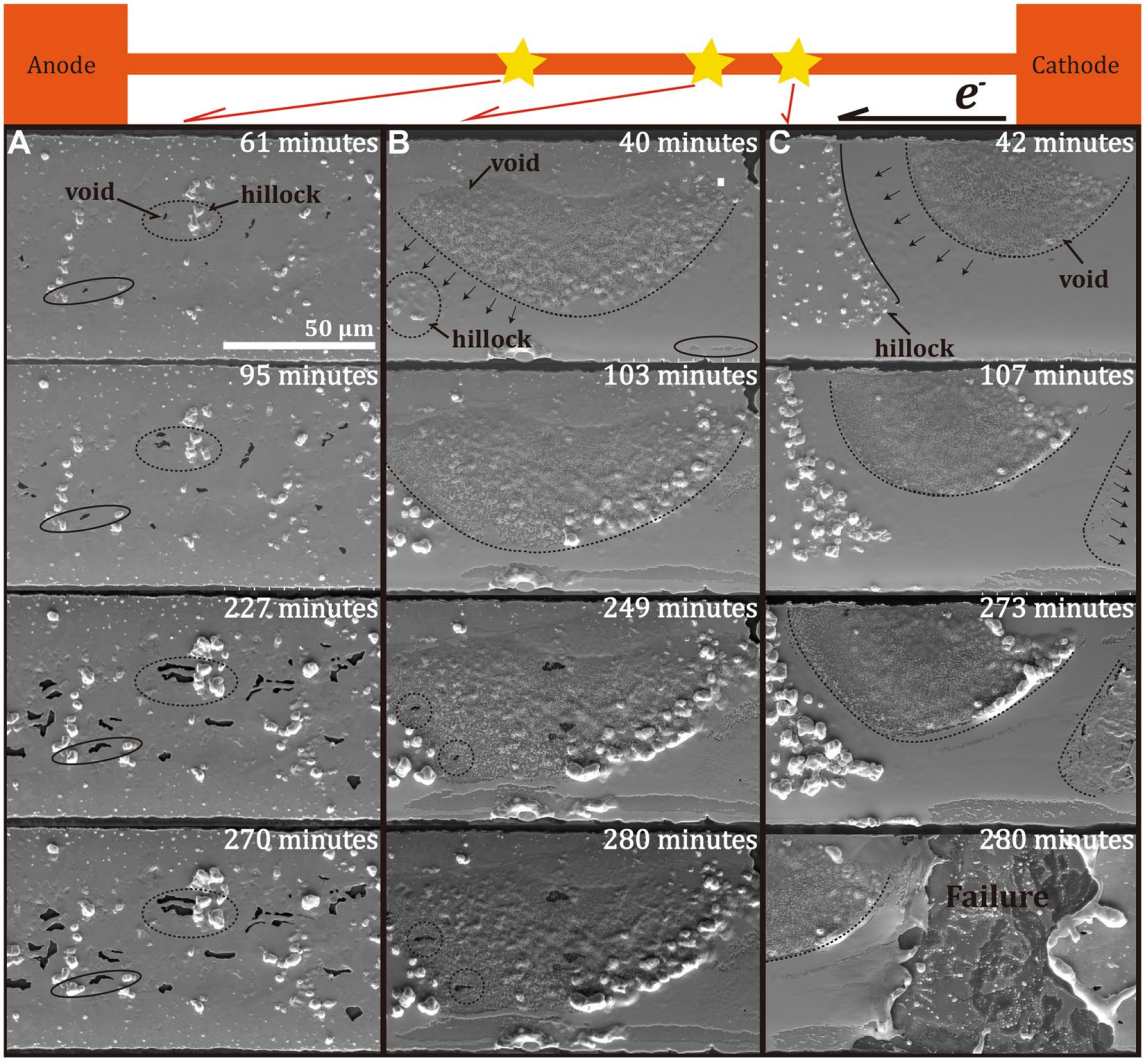
The morphology evolutions of the 80 μm-wide Cu strip under current stressing at the current density of 1.0 × 10^6^ A/cm^2^ at the positions indicted in the schematic diagram with points A, B and C (the videos of *in situ* morphology evolution are available in supplementary).

We further investigated the spatial distribution of the electron flow-induced plastic deformation along the Cu strips by taking the 80 μm-wide Cu strip with a current density of 1.0 × 10^6^ A/cm^2^ as an example using *in situ* SEM for various lengths of time until the strip was broken after 280 min. of current stressing (see Fig. S10 in supplementary for the as-prepared morphology). Figure 6 shows the morphology evolutions of the Cu strip at the positions near the middle and near-cathode, respectively, as indicated in the schematic diagram. In agreement with the *in situ* X-ray measurements, the most severe microstructural deformations occurred at around the middle (*m*) and the near cathode (*n-c*), where the largest electron flow-induced strains were detected. The Cu strip eventually failed at point C after 280 min. of current stressing. This finding also suggests the large electron flow-induced, greater than the “yield strain”, triggers the plastic deformations. In addition, the spatial distribution of the hillocks and anodes was not as expected according to conventional theories – formation of hillocks at the anode and voids at the anode. Instead, the voids and hillocks formed all along the strip, in accordance with the extent of electron flow-induced strains. Moreover, as shown in Fig. 6A, by tracking the hillock-void pairs (see supplementary for the videos of the microstructural evolution), the Cu atoms did not diffuse along the direction of the electron flow. Similarly, as shown in Fig. 6B and C, the propagation of voids did not follow the direction of electron flow. The results clearly indicate the “local stress relaxation” dominates the formation of hillocks and voids under current stressing. This result seems to contradict the experimental observations in the literature^1^; however, as discussed in the previous section, from a macroscopic point of view, a gradient of the electron flow-induced strains exists along the strip. The overall mass transport under current stressing, *i.e*., EM, results from the interplays of different extents of “local stress relaxation” along the metal strip. Hence, the *directional effect* of EM along with the flow of electrons can be observed, especially for experiments with short strips, *e.g*., the longest Al strips in Blech’s experiment^26^ was 150 μm, whereas the Cu strips in the present study are 2-cm long. Based on *in situ* and spatial microstructural evolutions, when the electron flow-induced strain is beyond the “yield strain”, the “local stress relaxation” is identified as the mechanism of hillock/void formation as well as the failure caused by EM.

### Mechanism of the electromigration effect

**Figure 7:**
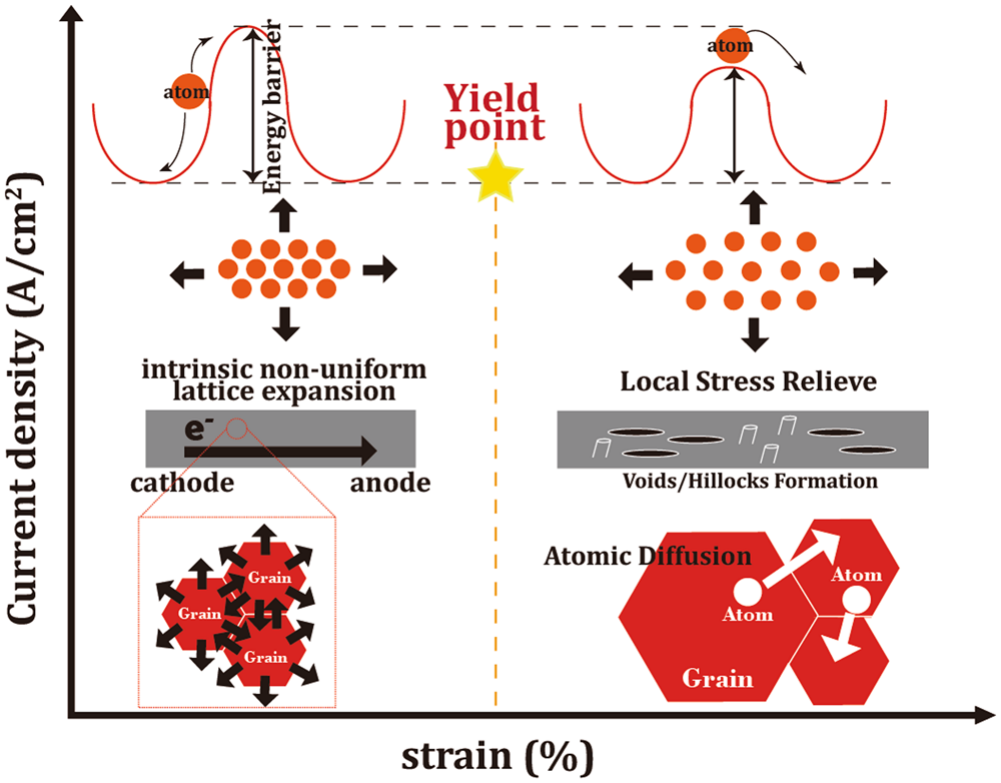
The schematic diagram of the universal EM mechanism proposed in this work.

Figure 7 summarizes the universal EM mechanism proposed in this work. When electrons flow through a Cu strip, a non-uniform electron flow-induced strain will be established from the cathode toward the anode. Higher current density will induce a larger strain. As shown in Fig. 7, the microstructural responses of Cu against current stressing can be categorized into two regions: (1) The elastic deformation region: When the current density is smaller than the threshold value, that its induced strain is lower than the “yield strain”, the Cu atoms are trapped in the energy well of lattice sites and the lattice expansion can be recovered reversibly after the cessation of current stressing; and (2) the plastic deformation region: When the current density is sufficiently high, that the electron flow-induced strain is large enough, exceeding the “yield strain”, the diffusion Cu atoms is activated owing to the reduced barrier between neighboring lattice sites under tension, and the formation of hillocks and voids will occur as a result of local stress relaxation. This proposed mechanism of EM is universal and can be employed to comprehend the experimental observations in the literatures as follows:

(1) EM was enhanced for materials under tension but retarded by compression^27^. This is because the electron flow-induced strains lead to a tension condition immediately after the current stressing. The pre-existing tension stress in the materials exacerbates the total tensile strain to exceed the yield point, resulting in plastic deformation and the occurrence of EM-induced failures. In contrast, the pre-existing compressive stress in materials helps hinder the total tensile strain from the yield point. (2) Adding rigid cap-layers on metal strips delayed the occurrence of EM^26, 28, 29^, while if the cap-layers were not fully covered^26^ or not well contacted^30^, the decelerating of EM-induced failure became invalid. This phenomenon can also be comprehended based on the proposed mechanism. If a material were fully covered by rigid walls, the lattice expansion of the material under current stressing is suppressed; therefore, the rigid cap-layers can either postpone the time to reach the yield point or increase the threshold current density for yielding. (3) Stronger materials, *e.g*., (111)-textured^31–34^, precipitation hardened^33, 35, 36^, and nanotwin-modified^37^ materials, showed larger resistances to EM-induced failure. It can be rationalized based on the proposed mechanism that the stiffer materials have stronger resistances to electron flow-induced expansion, so they are more prone to being retained in the elastic region shown in Fig. 7 without the occurrence of EM under current stressing. On top of this universal mechanism, it must be noted that materials with fine grains possess enhanced mechanical strength^37^; however, if the mechanical property was not enhanced considerably and the electron flow-induced strain exceeds the yield point, the large amount of grain boundaries helps the local stress relaxation and dramatically accelerates the failure due to rapid diffusion paths^38^. For such a case, the EM-lifetime will instead decay when reducing the grain size. The competing effects of reduced grain sizes on enhancing the mechanical property and promoting the rapid grain boundary diffusion path should be considered.

## Conclusions

In this paper, current stressing experiments on pure Cu strips using *in situ* synchrotron XRD and *in situ* SEM measurements and corresponding *ab initio* calculations and finite-element simulations were performed. A non-uniform lattice expansion induced intrinsically by the flow of electrons alone is identified – larger at the cathode and smaller at the anode, establishing a tensile strain gradient along the flow of electrons. *Ab initio* calculations agree closely with the experimental findings that the electron perturbation, *i.e*., electron injection and ejection, involves a net lattice expansion. This electron flow-induced strain is proportional to the exerted current density. When the current density is low, the electron flow-induced strain is an elastic deformation and the lattice will recover following the cessation of electron flows; however, when the current density is sufficiently high, the electron flow-induced strain will exceed the yield strain and initiate diffusional creep as local stress relaxation, causing the formation of hillocks and voids as well as EM-induced failure. The fundamental driving force for the electromigration effect is elucidated and can be analogized with conventional solid mechanics.

## Electronic supplementary material

Electronic Supplementary Information

Video S1

Video S2

Video S3
