# Supplementary material for: The electromigration effect revisited: non-uniform local tensile stress-driven diffusion
Source: Sci Rep. 2017 Jun 8;7:3082. doi: 10.1038/s41598-017-03324-5 (PMC5465215; doi:10.1038/s41598-017-03324-5)
Supplement: Supplementary file 1 — Electronic Supplementary Information [file 41598_2017_3324_MOESM1_ESM.pdf]

Electronic Supplementary Information for

The electromigration effect revisited:

non-uniform local tensile stress-driven diffusion

Shih-kang Lin*, Yu-chen Liu, Shang-Jui Chiu, Yen-Ting Liu, and Hsin-Yi Lee

*To whom correspondence should be addressed. E-mail: linsk@mail.ncku.edu.tw (S.K.L.).

This PDF file includes:

Figures S1 to S10

Tables S1

Videos S1 to S3

Supplementary text

1. Crystal structure of the as-prepared pure Cu specimens

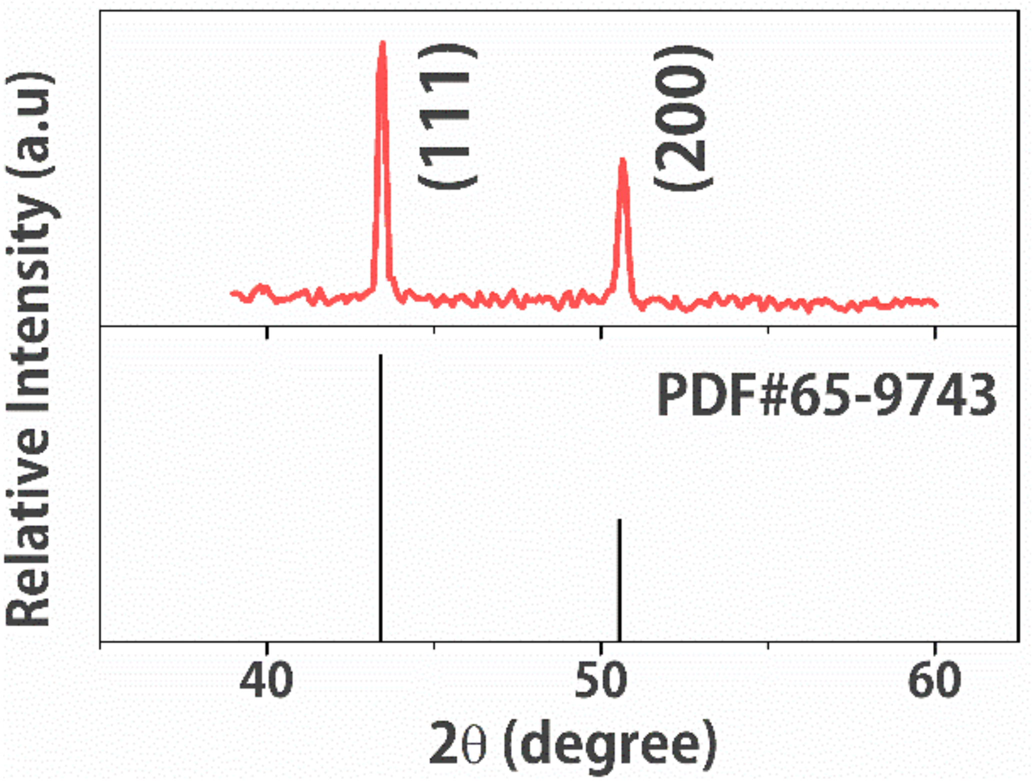

Figure S1: The XRD pattern of Cu dog-bone specimen annealed at 400 °C under N_2 atmosphere for 2 h and the standard pattern based on the JCPDS database (PDF #65-9743).

2. Experimental setups of *in situ* experiments

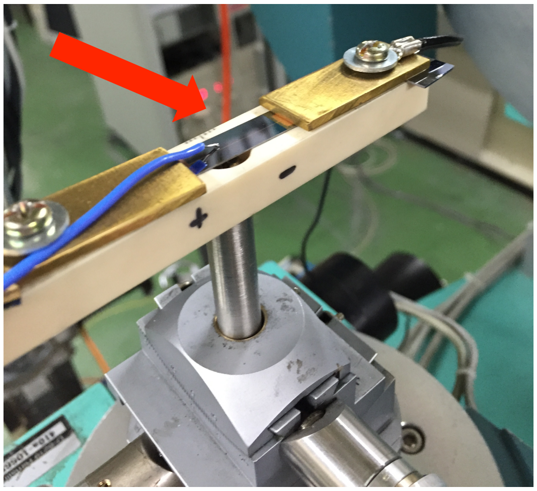 (A) 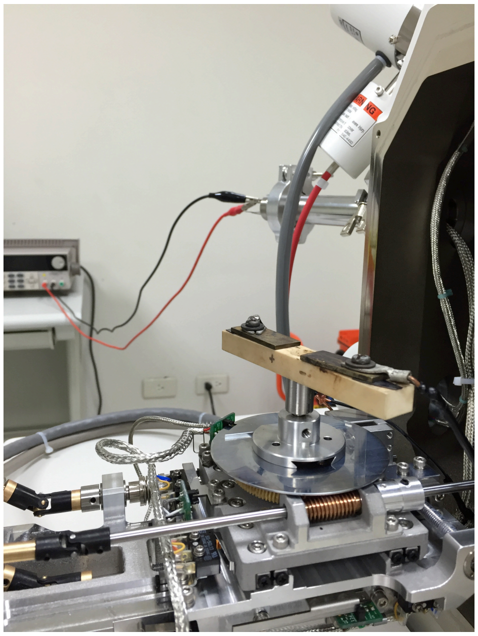 (B)

Figure S2. The experimental setup of the current-stressing experiments: (A) *in situ* synchrotron XRD and (B) *in situ* SEM. In (A), the red arrow indicates the X-ray incident beam, and a *K*-type thermocouple was attached at the middle side of the sample.

3. Derivation of the electron flow-induced strain

The "Middle" of 2 cm-long Cu strips with various widths of 50, 80, 100, and 500 μm were characterized under current stressing using *in situ* synchrotron XRD. The resultant evolutions of Cu-(111) peak for the 50, 80, and 100 μm-wide Cu strips under current stressing with current densities are shown in Figs. S3 – S5, respectively. These peaks were fitted to obtain systematic peak evolution using the *general model Gauss1* in Matlab software, *i.e.*, $f(x) = a_1 \cdot \exp\left[-\left(\frac{x-b_1}{c_1}\right)^2\right]$, where a_1, b_1 and c_1 are the fitting parameters. A high R-square of 99.54% was obtained, indicating good fitting quality. After fitting, the d-spacing of each peaks was calculated according to Bragg's Law, *i.e.*, $2d\sin\theta = \lambda$. Total strains were calculated by using $\epsilon = \frac{d-d_0}{d_0} \times 100\%$, where d and d_0 are the measured and equilibrium d-spacing, respectively, as shown in Figs. S6 – S8. The true electron flow induced-true strains were derived by using the temperature profiles of Cu strips under current stressing shown in Figs. S6F, S7F and S8E together with the measured thermal strains shown in Fig. 2C. The true electron flow induced-true strains and all the pertinent data are tabulated in Table S1.

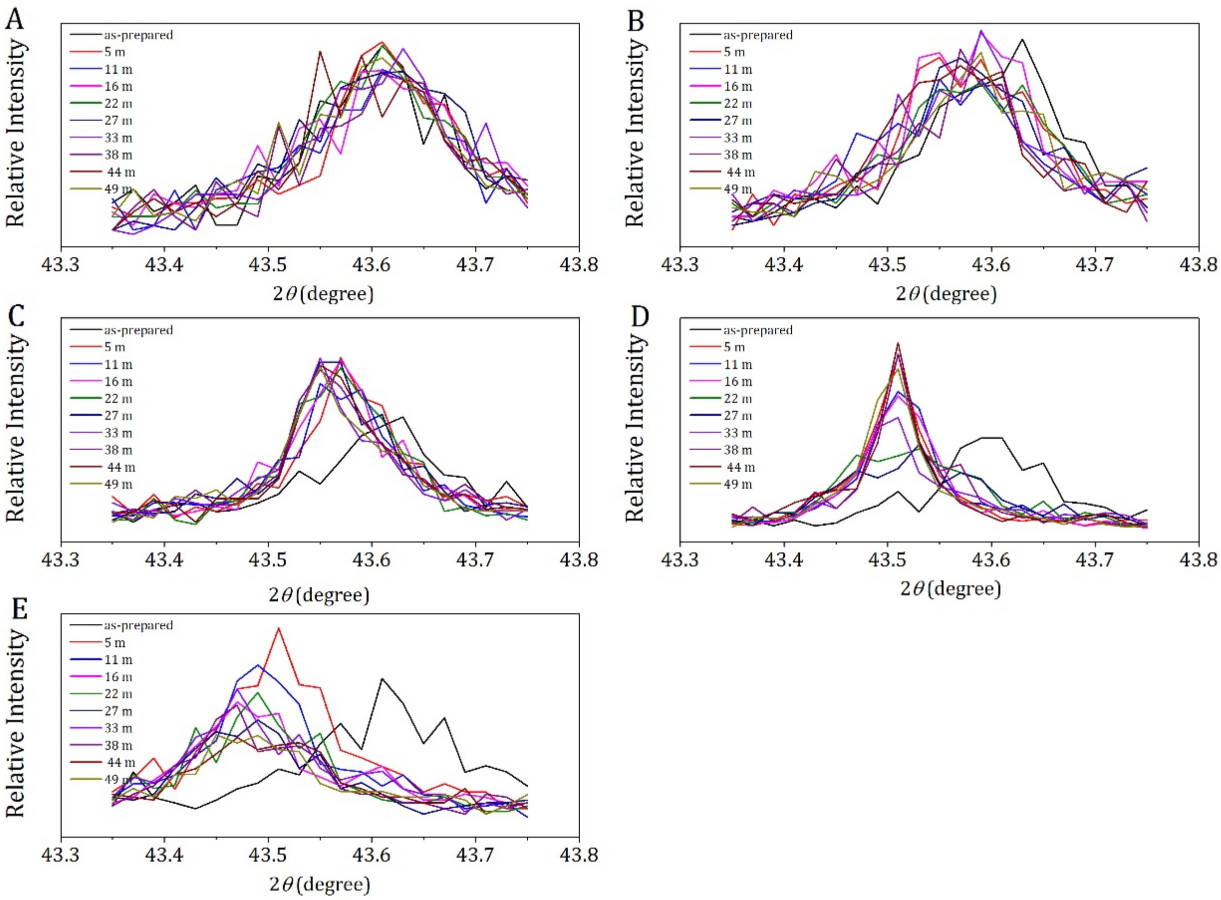

Figure S3: The XRD peaks of Cu-(111) of 50 μm-wide strips under current stressing with current density of (A) 2.0 x 10^5, (B) 6.0 x 10^5, (C) 8.0 x 10^5, (D) 10.0 x 10^5, and (E) 12.0 x 10^5 A/cm^2.

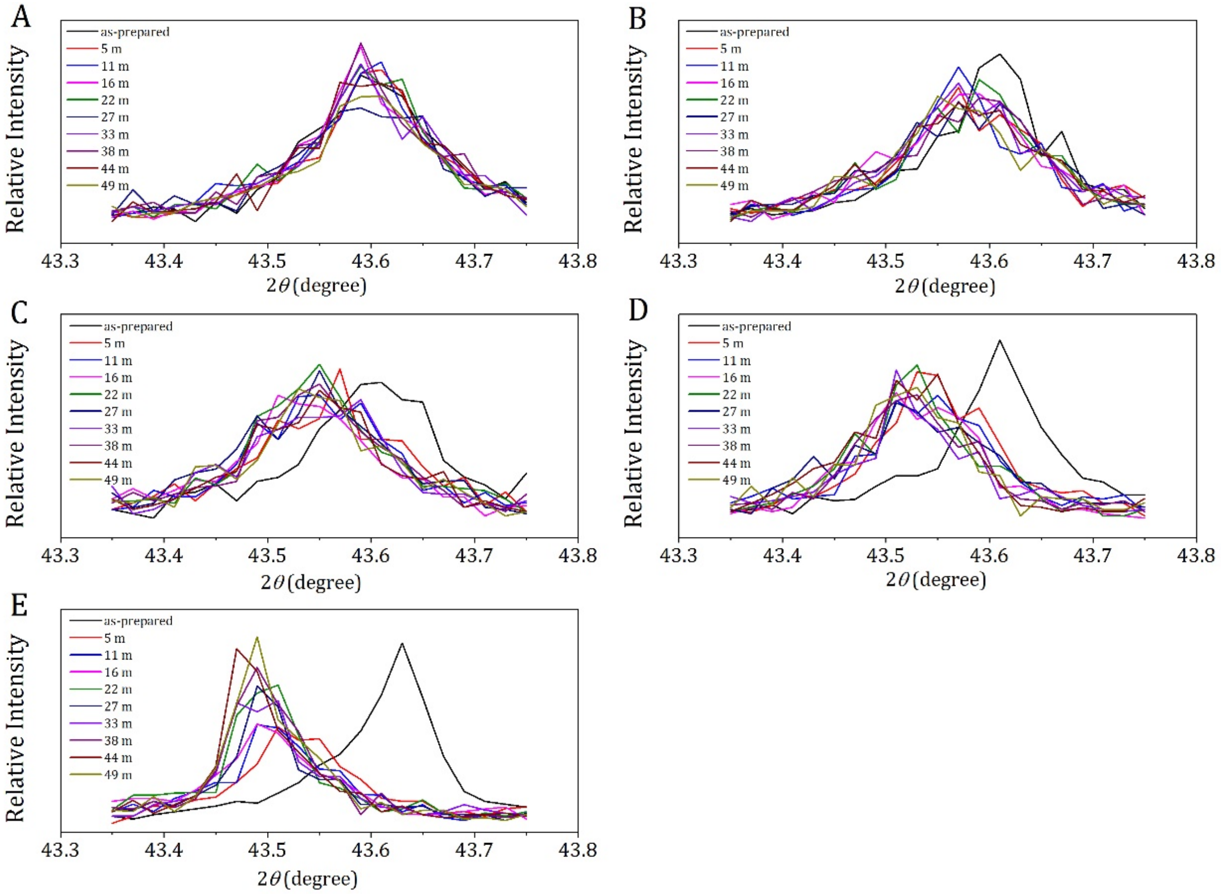

Figure S4: The XRD peaks of Cu-(111) of 80 μm-wide strips under current stressing with current density of (A) 2.5 x 10^5, (B) 5.0 x 10^5, (C) 7.5 x 10^5, (D) 8.75 x 10^5, and (E) 10.0 x 10^5 A/cm^2.

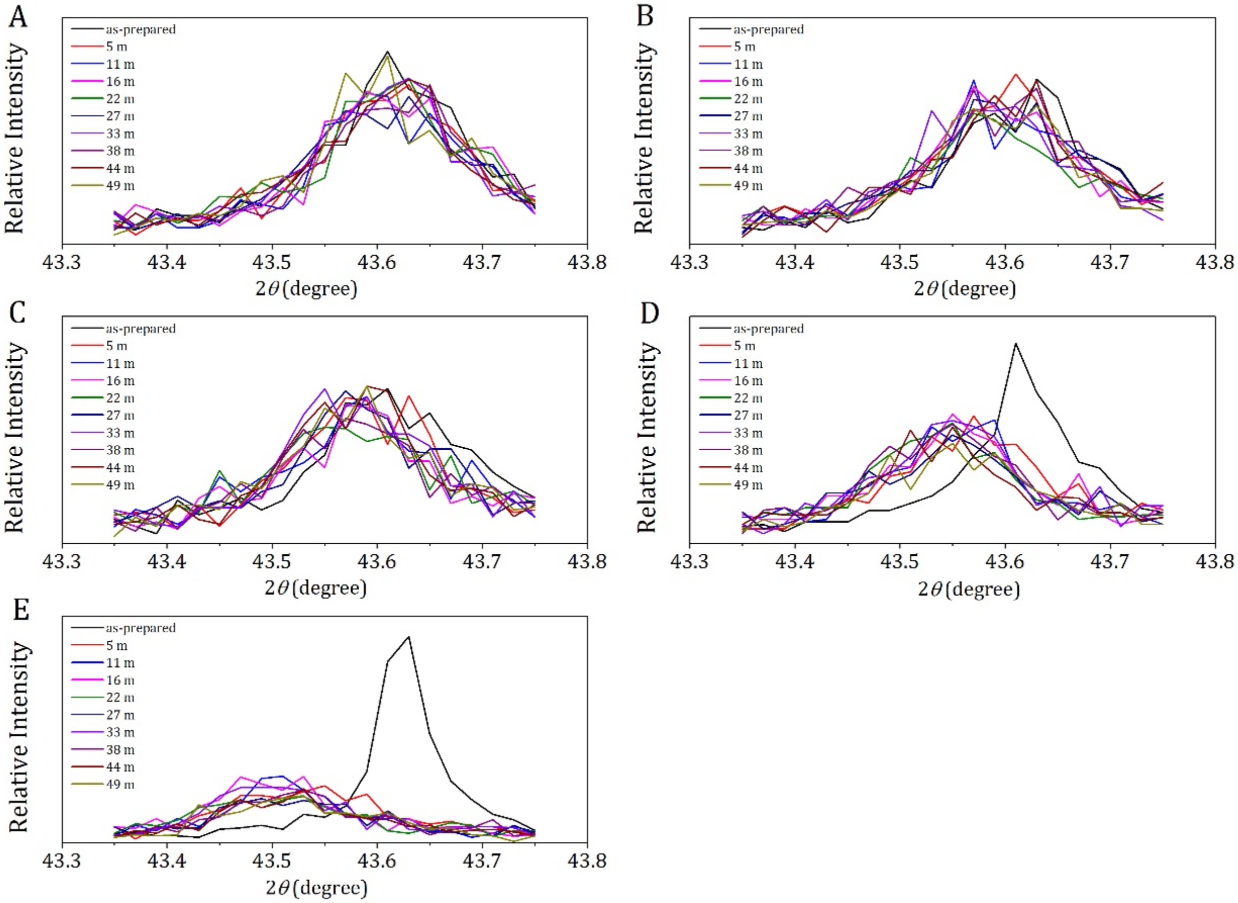

Figure S5: The XRD peaks of Cu-(111) of 100 μm-wide strips under current stressing with current density of (A) 2.0 x 10^5, (B) 4.0 x 10^5, (C) 6.0 x 10^5, and (D) 8.0 x 10^5 A/cm^2.

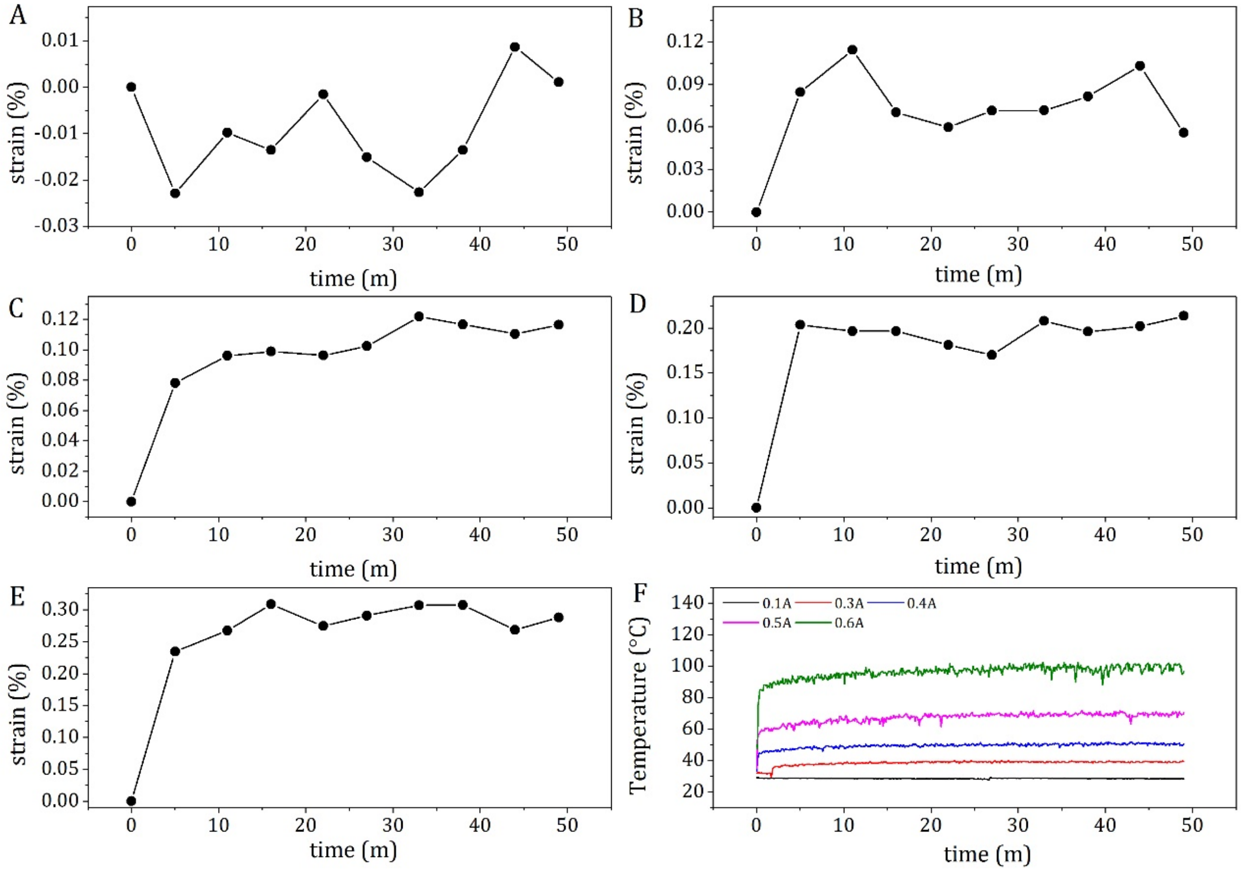

Figure S6: The total strains of 50 μm-wide Cu strips under current stressing with current density of (A) 2.0 x 10^5, (B) 6.0 x 10^5, (C) 8.0 x 10^5, (D) 10.0 x 10^5, and (E) 12.0 x 10^5 A/cm^2. (F) The temperature profile of 50 μm-wide Cu strips under current stressing under current stressing.

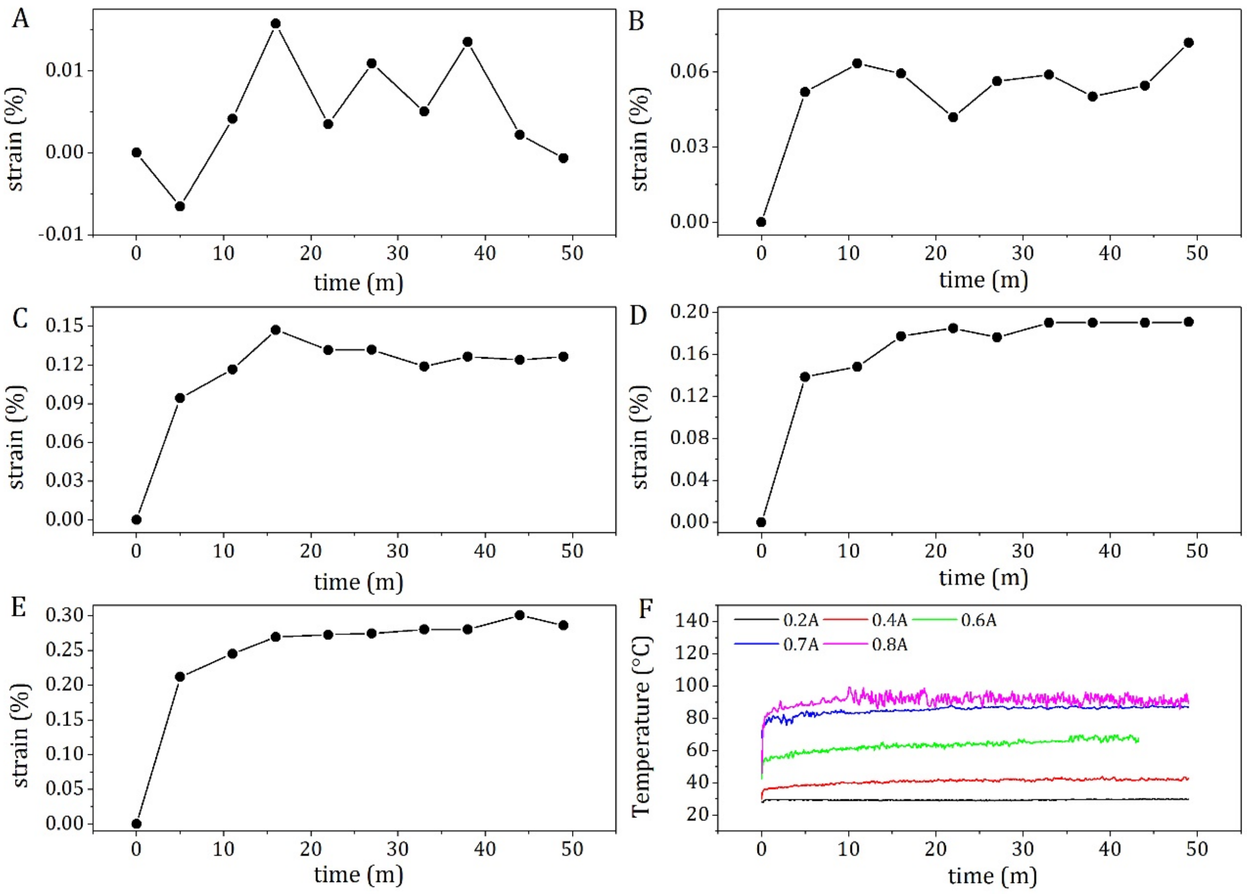

Figure S7: The total strains of 80 μm-wide Cu strips under current stressing with current density of (A) 2.5 x 10^5, (B) 5.0 x 10^5, (C) 7.5 x 10^5, (D) 8.75 x 10^5, and (E) 10.0 x 10^5 A/cm^2. (F) The temperature profile of 80 μm-wide Cu strips under current stressing under current stressing.

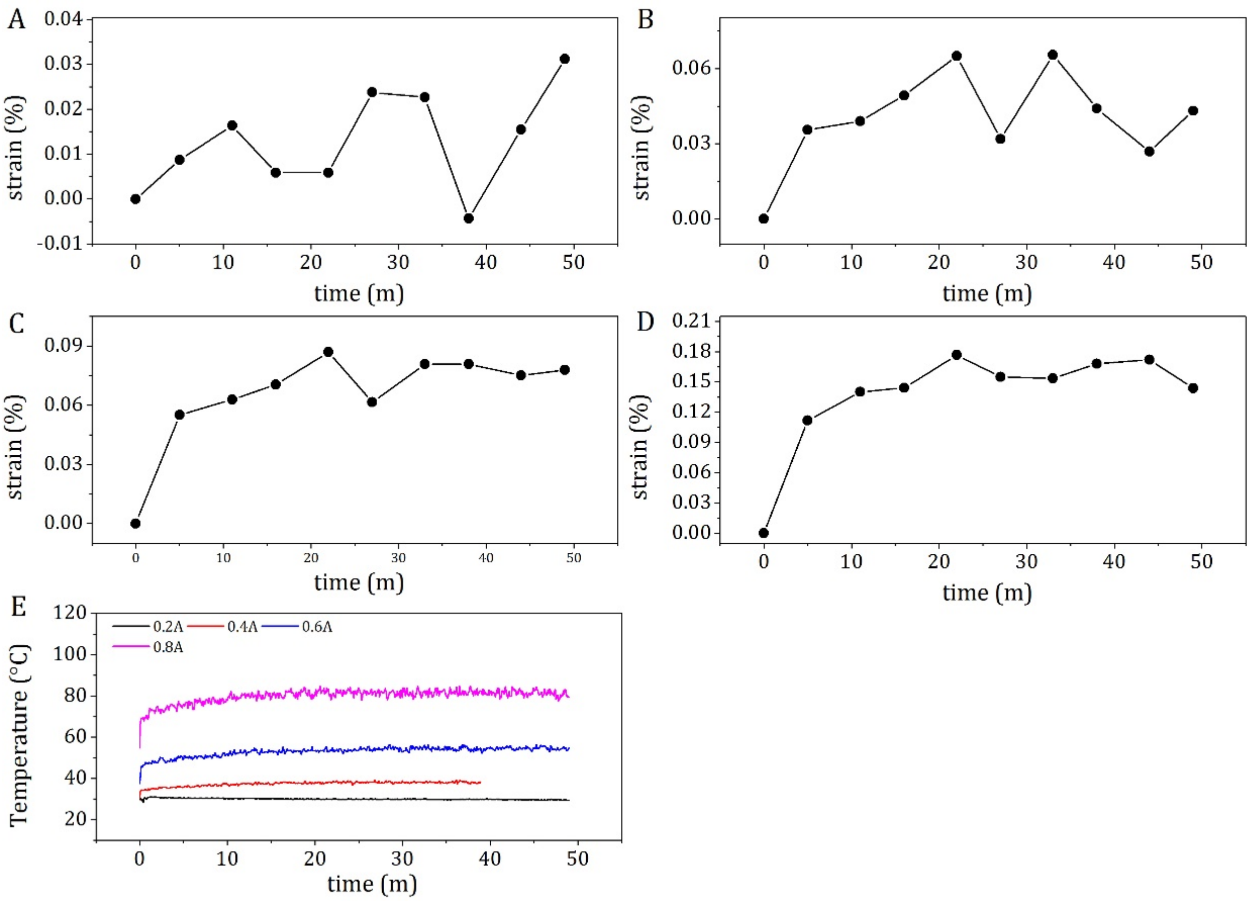

Figure S8: The total strains of 100 μm-wide Cu strips under current stressing with current density of (A) 2.0 x 10^5, (B) 4.0 x 10^5, (C) 6.0 x 10^5, and (D) 8.0 x 10^5 A/cm^2. (E) The temperature profile of 100 μm-wide Cu strips under current stressing.

Table S1. The total strains derived from *in situ* synchrotron XRD, the equilibrium temperatures, the derived heat-induced strains, and electron flow-induced strains for the Cu strips with various widths under current stressing at various current densities.

Width (μm)	Current density (x10^5 A/cm^2)	Total Strain (%)	Temperature (^{o}C)	Heat-induced strain (%)	Electron flow -induced strain (%)
50	2.0	-0.007	28.43	0.000	-0.007
	6.0	0.078	39.03	0.003	0.075
	8.0	0.116	50.47	0.033	0.083
	10.0	0.205	69.35	0.084	0.121
	12.0	0.293	98.59	0.160	0.132
80	2.5	0.005	29.59	0.000	0.005
	5.0	0.059	42.08	0.012	0.047
	7.5	0.124	67.31	0.078	0.046
	8.75	0.190	86.78	0.128	0.062
	10.0	0.287	91.21	0.137	0.150
100	2.0	0.016	29.73	0.000	0.016
	4.0	0.045	38.31	0.001	0.044
	6.0	0.079	54.51	0.044	0.035
	8.0	0.160	81.55	0.114	0.045
500	4.0	0.282	109.39	0.188	0.083

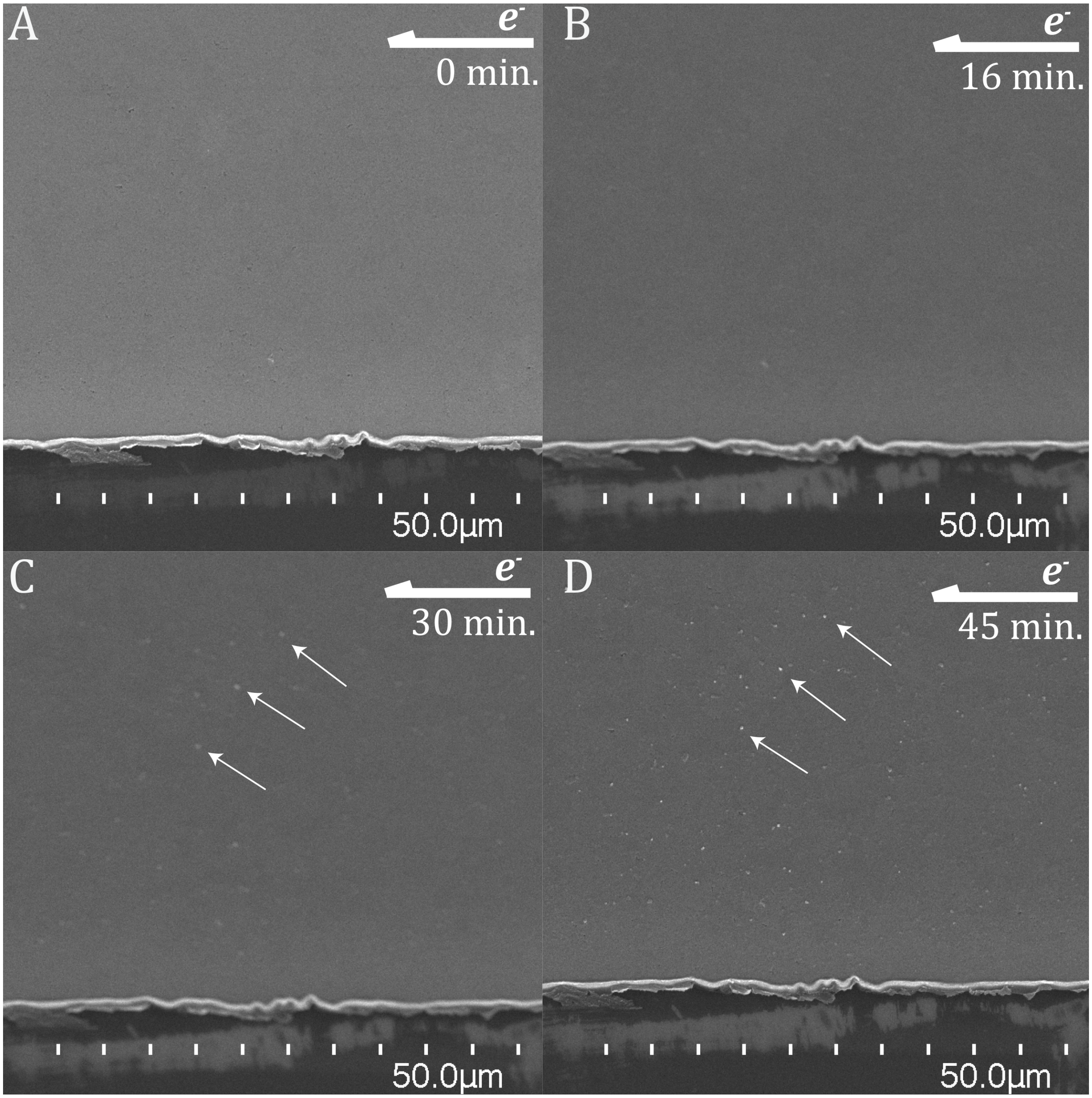

Figure S9: The morphology evolution of a 80 μm-wide Cu strip under current stressing at the current density of 7.5×10^5 A/cm^2 for (A) 0, (B) 16, (C) 30, and (D) 45 min. The white arrows indicate the formation of small hillocks at early stage.

4. *In situ* SEM morphology under electric current

Figure S10 shows the morphology of the as-prepared 80 μm-wide Cu strip: at "anode" (*a*), "near-anode" (*n-a*), "middle" (*m*), "near-cathode" (*n-c*), and "cathode" (*c*). No defects can be found prior to the current-stressing experiments.

In situ formation of voids and hillocks was recorded, and the evolutions are shown in the videos, available online at the electronic supplementary information. Videos S1 to S3 are the morphology evolution at the positions A, B, and C, respectively, indicted in the schematic diagram of Fig. 6 in the manuscript.

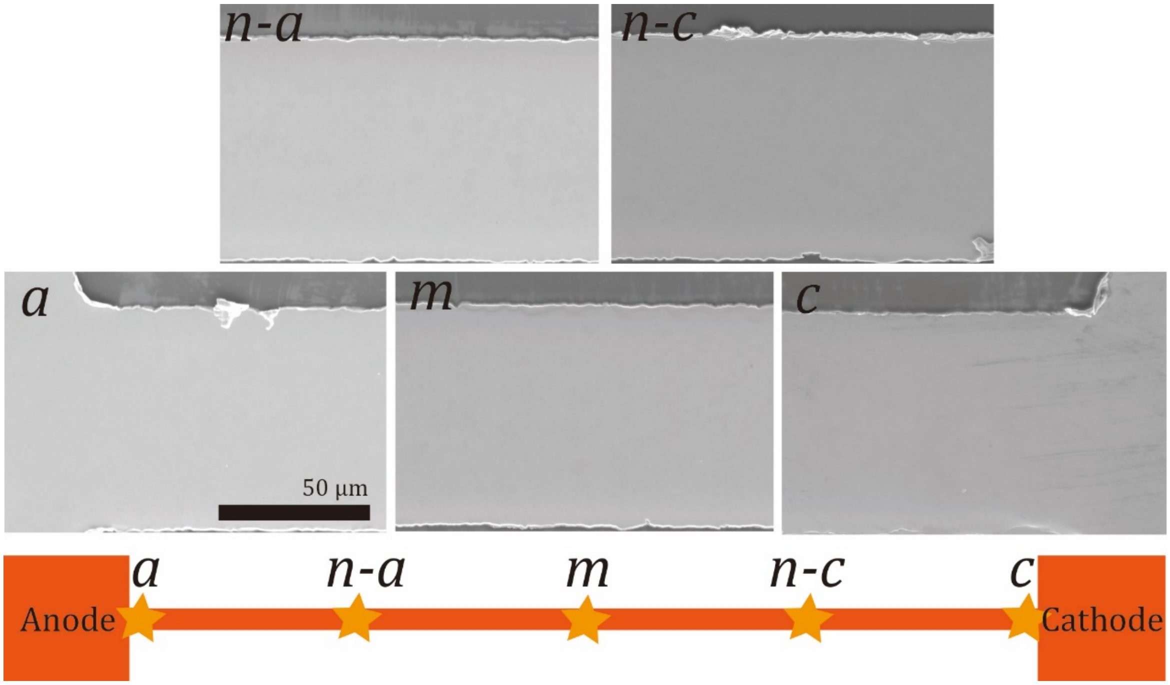

Figure S10. As-prepared 80-μm-width Cu strip morphology at "anode", "near-anode", "middle", "near-cathode", and "cathode" side. No apparent cracks could be observed.
