# Supplementary figures and images for: The electromigration effect revisited: non-uniform local tensile stress-driven diffusion
Source: Sci Rep. 2017 Jun 8;7:3082. doi: 10.1038/s41598-017-03324-5 (PMC5465215; doi:10.1038/s41598-017-03324-5)

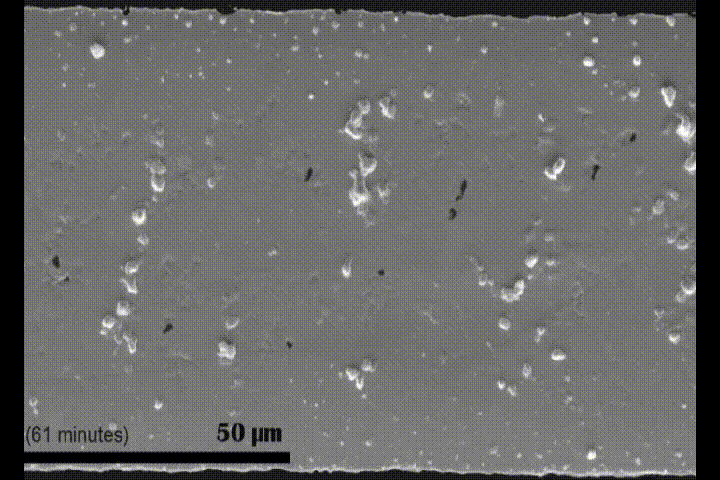

Supplement: Supplementary file 2 — Video S1 [file 41598_2017_3324_MOESM2_ESM.gif]

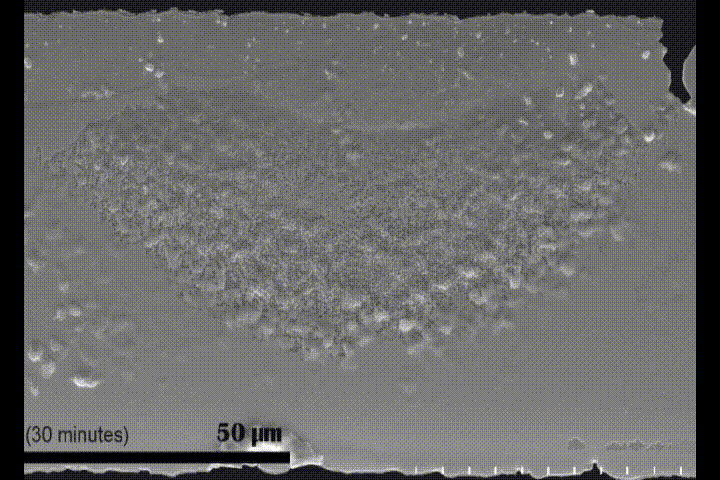

Supplement: Supplementary file 3 — Video S2 [file 41598_2017_3324_MOESM3_ESM.gif]

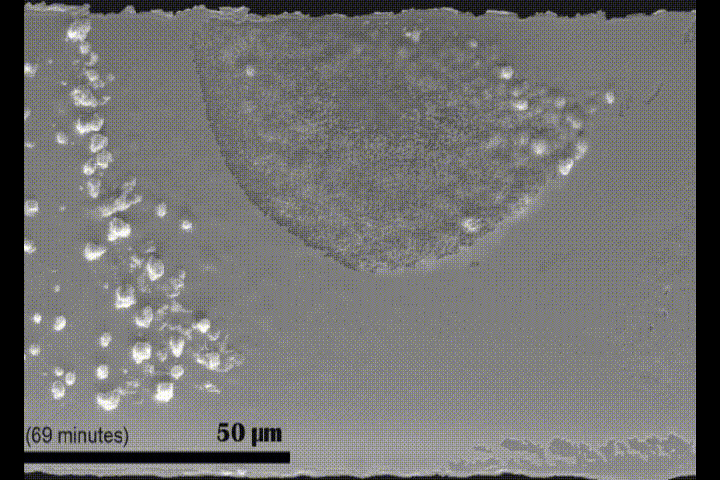

Supplement: Supplementary file 4 — Video S3 [file 41598_2017_3324_MOESM4_ESM.gif]
